# Impact of acute undernutrition on growth, ileal morphology and nutrient transport in a murine model

**DOI:** 10.1590/1414-431X20165340

**Published:** 2016-10-10

**Authors:** I.C. Sampaio, P.H.Q.S. Medeiros, F.A.P. Rodrigues, P.A. Cavalcante, S.A. Ribeiro, J.S. Oliveira, M.M.G. Prata, D.V.S. Costa, S.G.C. Fonseca, M.M. Guedes, A.M. Soares, G.A.C. Brito, A. Havt, S.R. Moore, A.A.M. Lima

**Affiliations:** 1 Departamento de Fisiologia e Farmacologia, Faculdade de Medicina, Instituto de Biomedicina, Universidade Federal do Ceará, Fortaleza, CE, Brasil; 2 Departamento de Farmácia, Universidade Federal do Ceará, Fortaleza, CE, Brasil; 3 Division of Gastroenterology, Hepatology, and Nutrition, Cincinnati Children's Hospital Medical Center, Cincinnati, OH, USA

**Keywords:** Undernutrition, Ion transport, Intestinal absorption

## Abstract

Undernutrition represents a major public health challenge for middle- and low-income countries. This study aimed to evaluate whether a multideficient Northeast Brazil regional basic diet (RBD) induces acute morphological and functional changes in the ileum of mice. Swiss mice (∼25 g) were allocated into two groups: i) control mice were fed a standard diet and II) undernourished mice were fed the RBD. After 7 days, mice were killed and the ileum collected for evaluation of electrophysiological parameters (Ussing chambers), transcription (RT-qPCR) and protein expression (western blotting) of intestinal transporters and tight junctions. Body weight gain was significantly decreased in the undernourished group, which also showed decreased crypt depth but no alterations in villus height. Electrophysiology measurements showed a reduced basal short circuit current (*I*_sc_) in the undernourished group, with no differences in transepithelial resistance. Specific substrate-evoked *I*_sc_ related to affinity and efficacy (glutamine and alanyl-glutamine) were not different between groups, except for the maximum *I*_sc_ (efficacy) induced by glucose. Transcription of *Sglt1* and *Pept1* was significantly higher in the undernourished group, while *SN-2* transcription was decreased. No changes were found in transcription of CAT-1 and CFTR, while claudin-2 and occludin transcriptions were significantly increased in the undernourished group. Despite mRNA changes, SGLT-1, PEPT-1, claudin-2 and occludin protein expression showed no difference between groups. These results demonstrate early effects of the RBD on mice, which include reduced body weight and crypt depth in the absence of significant alterations to villus morphology, intestinal transporters and tight junction expression.

## Introduction

Undernutrition is defined as a physiological outcome of illness and/or hunger, which subclassifies as wasting (an acute state), stunting (a chronic state), underweight (mixed acute and/or chronic states) and micronutrient deficiencies. This condition has a great impact on global public health, especially in middle- and low-income countries (1,2), and is the cause of 3.1 million child deaths annually. Further, it is estimated that 25.7% (about 165 million) of children younger than 5 years suffer from stunting, while 10.9% (∼70 million) and 15.7% (∼100 million) suffer from wasting and underweight, respectively (3). Further evaluation of childhood undernutrition-associated consequences has worrisome social implications, such as impaired school performance (4), increased economic costs (5), impaired immunity (6) and a significantly higher number of deaths due to infectious diseases (7).

The regional basic diet (RBD) is an experimental rodent diet based on the nutritional intake of the northeastern Brazilian population and is characterized by a deficit of protein, fat and minerals, which trigger some clinical symptoms of kwashiorkor and stunting, commonly reported in this population (8,9). Some studies have used the RBD in mouse models of undernutrition (10–12). Chronically, the RBD promotes deleterious effects in the small intestine with altered villous height and crypt depth, reduced transmucosal resistance, increased permeability and enhanced epithelial apoptosis (12).

Several studies seek to understand the pathophysiology of undernutrition by characterizing alterations of transcellular and paracellular transports (12–19). Transmembrane proteins, such as sodium-glucose linked transporter 1 (SGLT-1), peptide transporter 1 (PEPT-1) and cationic amino acid transport-1 (CAT-1), are modulated by undernourished states (14–16,19). Conversely, the paracellular transport mediating tight junctions, such as claudins, occludins and zonula occludens, have been described as fundamental for absorption of electrolytes and water in the small intestine, and are modulated by undernutrition as well (12,13,17–19).

Generally, the clinical management of undernutrition requires early detection of pathophysiological changes. As the majority of studies have shown only the long-term effects of RBD, we examined if the RBD induces acute morphological and functional changes in the ileum in a short period of time, evaluating modifications of electrophysiological parameters and transcription and expression of intestinal transporters and tight junction proteins.

## Material and Methods

### Animals and experimental design

Male Swiss mice (total of 28) weighing ∼25 g were obtained from the Departamento de Fisiologia e Farmacologia, Universidade Federal of Ceará, and maintained under controlled temperature (21±2°C) and humidity (60±5%), and a 12:12 h light:dark cycle. Mice had access to a standard commercial diet and water *ad libitum*. The animal protocols were performed according to the norms of the National Council for Control of Animal Experimentation (CONCEA) and were approved by the Ethics Committee of Animal Research of the Universidade Federal do Ceará, Fortaleza, CE, Brazil (protocol #62/11).

**Figure 1:**
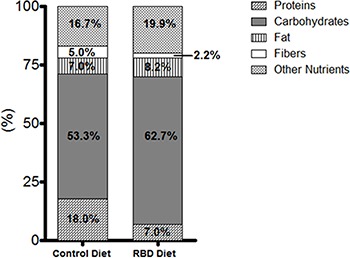
Composition of the regional basic diet (RBD) and control diet reported as percent of total calories. The RBD diet contains a higher amount of carbohydrates and lower quantity of protein than the control diet.

Mice were divided randomly into two groups. The control group was fed a standard diet (Biotec^®^, Brazil) while the experimental undernourished group was fed the RBD diet. The RBD diet was developed at the Laboratório de Farmacotécnica, Universidade Federal do Ceará, as previously described (8,9). The composition of these diets is shown in Figure 1.

During the experimental period, animals were maintained in metabolic cages. Weight, and food and water intake were measured daily. After seven days of RBD feeding, electrophysiological parameters, intestinal morphometry, gene transcription and expression of intestinal transporters and tight junction proteins were evaluated from ileal segments. After anesthetizing animals with 10 mg/kg xylazin and 90 mg/kg ketamine, intestinal segments were collected and major blood vessels ligated to induce exsanguination.

### Ussing chambers system

We evaluated the transport of glucose, glutamine and alanyl-glutamine by Ussing chambers as previously described (12). Eight-centimeter ileal segments were collected, quickly dissected from serous membrane, opened along the mesenteric attachment remnant and cut into 1.5 cm pieces inside Petri dishes containing Krebs solution. The tissue was immediately mounted in Ussing chambers with the mucosal side faced up on the hemi-chamber (WPI, USA). For each experiment, the average tissue loss was less than 20%. Each assay was performed with 2 mice; one undernourished and one control, from which four ileal segments were collected. The tissue was perfused with Krebs solution (10 mM glucose in serosal and 10 mM mannitol in mucosa) containing 115 mM NaCl, 25 mM NaHCO_3_, 2.4 mM K_2_HPO_4_, 1.2 mM CaCl_2_, 1.2 mM MgCl_2_, and 0.4 mM KH_2_PO_4_, pH 7.4. This buffer was continuously gassed with 95% O_2_/5% CO_2_, and temperature was kept at 37°C using a circulating water pump controlled by a thermostat.

After a 30-min period for reaching a state of equilibrium, short circuit current (I_sc_) was measured before and after cumulative addition of glucose, glutamine or alanyl-glutamine at concentrations of 1, 2, 7, 20, 70, and 200 mM applied to the mucosal side. The osmolarity of these solutions was adjusted to 300 milliosmoles by adding Ringer solution. To calculate resistance, a constant 50 µA current was applied. This value and the elicited spontaneous potential difference (PD) were inserted as input variables into Ohm's law, and the result was multiplied by 20 for unit normalization, as follows: R (resistance) = PD/I_sc_. The electrical viability of intestinal samples was verified after each experiment by evaluating electrical response to the addition of theophylline (5 mM) to the serosal side of the tissue. This addition inhibits phosphodiesterase enzyme, leading to elevated AMPc and consequent increased chloride secretion, demonstrated by the increased I_sc_ (20).

### Intestinal morphology analyses

Ileal segments were fixed with paraformaldehyde, dehydrated with ascending 70, 80, 90, and 100% ethanol and processed in paraffin. The resulting blocks were sliced into 5-µm-thick sections, stained with hematoxylin and eosin (H&E), and observed under a light microscope (×400). The area of the villi and crypt depth were measured as previously described (21) using Image J software version 1.6.0 (National Institutes of Health, USA).

### Evaluation of gene transcription of intestinal transporters and tight junctions

Gene transcription of sodium-glucose linked transporter (SGLT-1), system N-transporter (SN-2), cationic amino acid transporter (CAT-1), peptide transporter 1 (PEPT-1), cystic fibrosis transmembrane conductance regulator (CFTR,) zonula occludens-1 (ZO-1), claudin-1, claudin-2, and occludin was determined by quantitative reverse transcription-polymerase chain reaction (qRT-PCR). Initially, total RNA was isolated from mucosal membrane of ileal segments using RNeasy mini Kit (Qiagen, Germany) according to the manufacturer's instructions. The cDNA was synthesized using iScript™ cDNA Synthesis Kit (Invitrogen, USA). The reference gene *peptidylprolyl isomerase A* was used for this experiment (22). The primers design was based on mRNA sequences obtained from the National Center for Biotechnology Information (http://www.ncbi.nlm.nih.gov; accessed on February 4, 2014).

The qRT-PCR reactions were performed in a final volume of 25 µL containing 12.5 µL of iQ SYBR green supermix (Bio-Rad, USA), 200 nM (each) primers, and 1 µL of cDNA from sample. All primers and conditions for qRT-PCR are shown in Table 1. To measure the specificity of the applied amplifications (i.e., to determine whether the formed products were specific for the tested genes), we performed a melting curve analysis in which the reaction temperature was increased 0.5°C every 15 s, beginning at the annealing temperature of the tested set of primers and ending at 95°C. Throughout the curve construction process, the changes in fluorescence were measured, and the data obtained, using CFX Manager software (version 3.0; Bio-Rad), were based on the values for the threshold cycle, i.e., where the observed fluorescence was 10-fold higher than the basal fluorescence for each reaction. Gene transcription was obtained by applying the mathematical 2^-ΔΔ^CT method (23).

**Table:**
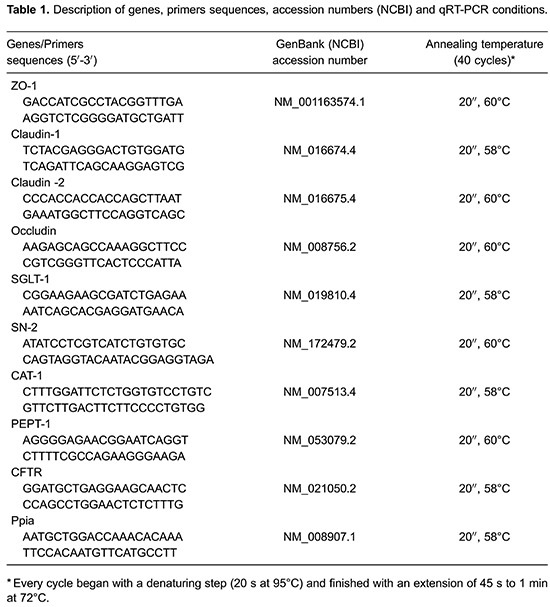

### Immunoblot analysis

In order to quantify SGLT-1, PEPT-1, claudin-2 and occludin proteins, mucosal membranes from ileal segments were homogenized in RIPA lysis buffer (25 mM Tris-HCL, pH 7.6; 150 mM NaCl; 5 mM EDTA; 1% NP40; 1% triton X-100; 1% sodium deoxycholate; 0.1% SDS) and protease inhibitor (1 µL inhibitor: 100 µL RIPA). The homogenates were centrifuged (17,949 *g*, 17 min, 4°C), and the supernatant was collected. Protein concentrations were determined through the bicinchoninic acid assay using PierceTM BCA protein assay kit (Thermo Scientific, USA). The protein (20 µg) was prepared adding Laemmli sample buffer with β-mercaptoethanol and denatured at 95°C for 5 min, except for PEPT-1, SGLT-1 and occludin. Then, proteins were separated in a SDS-polyacrylamide gel (8% for occludin, SGLT-1 and PEPT-1 analysis and 12.5% for claudin analysis) under a condition of 120 volts and transferred to polyvinylidene difluoride (PVDF) membranes by electrophoresis for 2 h. PVDF membranes were blocked with 5% bovine serum albumin for 1 h and incubated overnight with primary rabbit antibody [anti-β actin (1:1000), anti-SGLT-1 (1:500), anti-PEPT-1 (1:200), anti-claudin-2 (1:100), or anti-occludin (1:1000); Santa Cruz Biotechnology, USA]. Chemiluminescent detection using Clarity Western ECL Substrate (Bio-Rad) was performed after incubation of the membrane with secondary antibody (1:1000) for 1 h. Finally, bands were captured using the ChemiDoc system (Bio-Rad). Densitometric quantification of bands was made using Image J software version 1.6.0 (National Institutes of Health).

### Statistical analysis

Data are reported as means±SE. One- or two-way analysis of variance (ANOVA) followed by the Bonferroni’s *post hoc* test were used for parametric data. Gene transcription data were evaluated by the Mann-Whitney test. Differences were considered to be statistically significant when P<0.05. Analysis was performed using GraphPad Prism 5.0 (GraphPad Software, USA).

## Results

### Impact of RBD diet for 7 days on body weight

**Figure 2:**
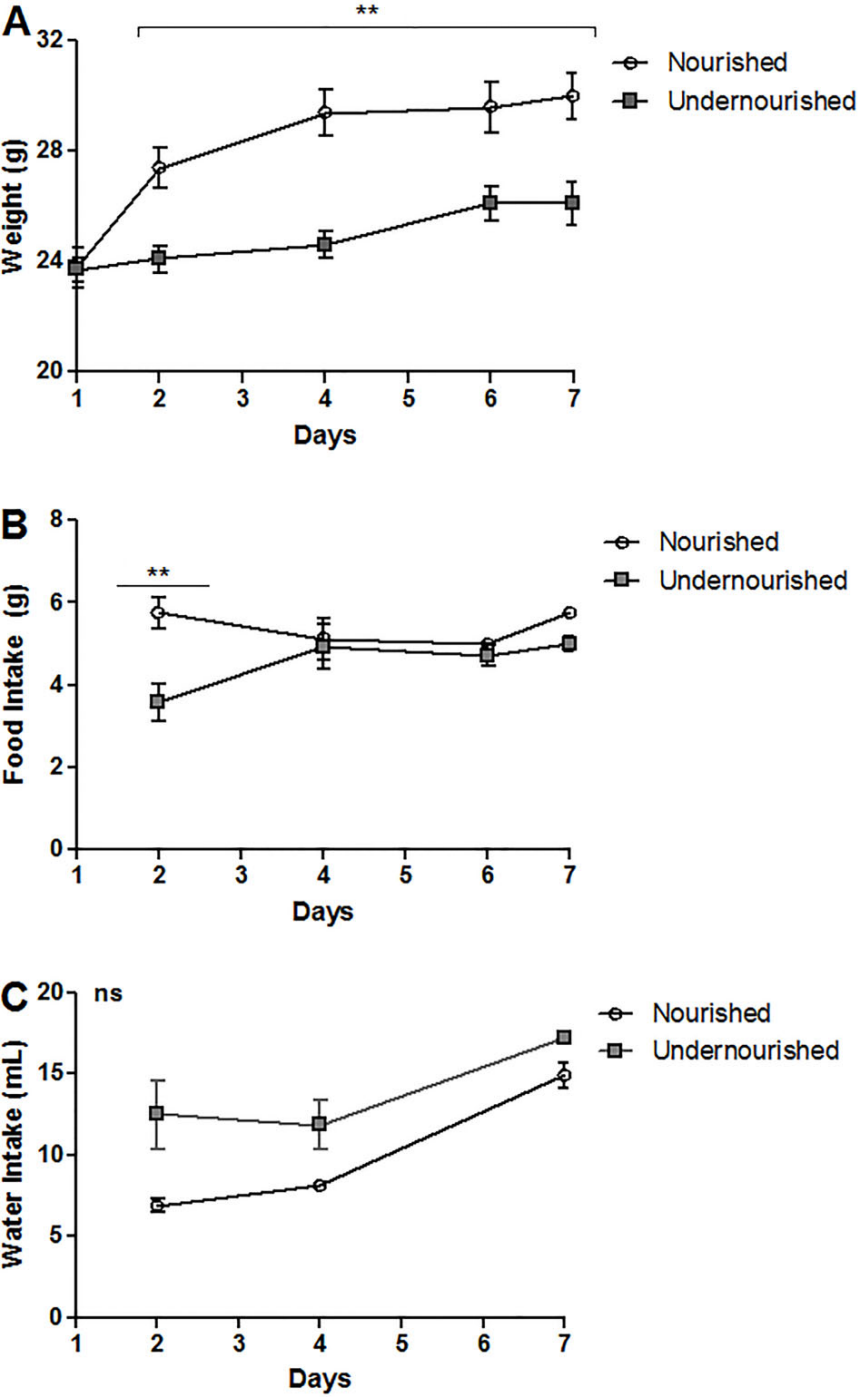
Weight curves from the regional basic diet (RBD) murine model of moderate acute undernutrition. The weight (*A*), food (*B*) and water (*C*) intake are shown after a 7-day challenge with the RBD. ns: non-significant. Data are reported as means±SE (n=4). **P<0.001 (ANOVA with the Bonferroni's multiple comparison test).

RBD feeding triggered a significant decrease in body weight gain when compared to the nourished group during the short period of 7 days (P<0.001). This difference was observable by the second day of intake, showing a reduction of 13.3% from the previous time point (nourished 30.0±0.8 *vs* 26.10±0.7 g undernourished; Figure 2A). In addition, food intake was higher in the nourished group until day 2 (P<0.01), and this difference was not observed subsequently (Figure 2B). Water intake did not differ statistically between the groups (Figure 2C).

### Impact of RBD diet for 7 days on intestinal morphology

**Figure 3:**
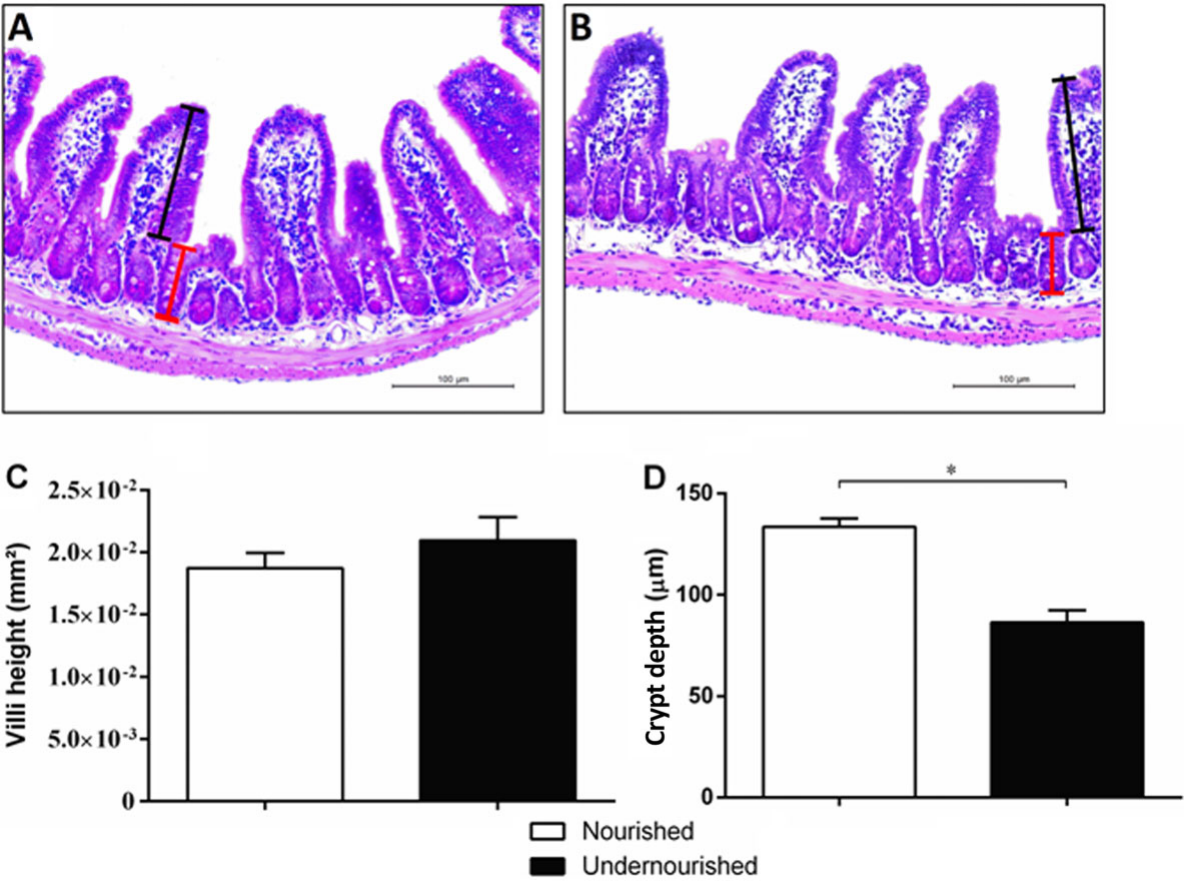
Photomicrographs and morphometric analysis of the ileum of nourished and undernourished mice. H&E-stained sections from nourished (*A*) and undernourished (*B*) mice. Crypt depth and villi height are represented, respectively, by red and black lines in each group (*A*, *B*). *C*, the index area of the villi and *D*, crypt depth from the ileum histological sections are shown for nourished and undernourished groups. Magnification 200×. Data are reported as means±SE (n=4). *P<0.01 (Mann-Whitney nonparametric test).

The intestinal morphometry of the ileum (Figure 3A and B) in the 7-days RBD diet group did not change with respect to the area of the villi compared to nourished control group (Figure 3C). Morphometric analysis showed a significant decrease in the depth of the crypts in the undernourished group compared to nourished control (133.6±4.12 *vs* 86.50±5.871 µm; P<0.001; Figure 3D).

### Impact of RBD diet for 7 days on basal short-circuit, electrogenic ion transport and tissue resistance

**Figure 4:**
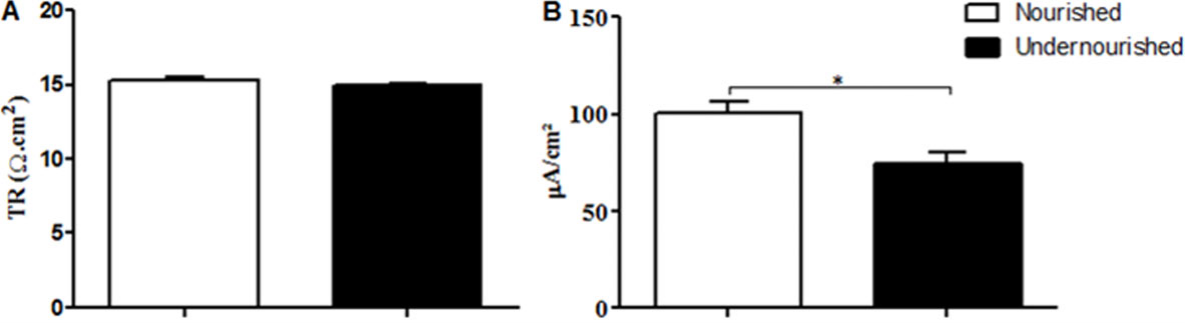
Comparison of transepithelial resistance (TR) (*A*) and basal short circuit current (*B*) for nourished and undernourished groups. Data are reported as means±SE (n=4). *P<0.05 (Mann-Whitney test).

In order to investigate whether intestinal ionic-substrate co-transport would be altered after 7 days of RBD, we measured basal I_sc_, electrogenic ion transports and transepithelial resistance of the ileum segment mounted in an Ussing chamber system. The basal I_sc_ was reduced significantly in the undernourished group compared to the nourished group (P=0.0297), while transepithelial resistance showed no difference (Figure 4).

**Figure 5:**
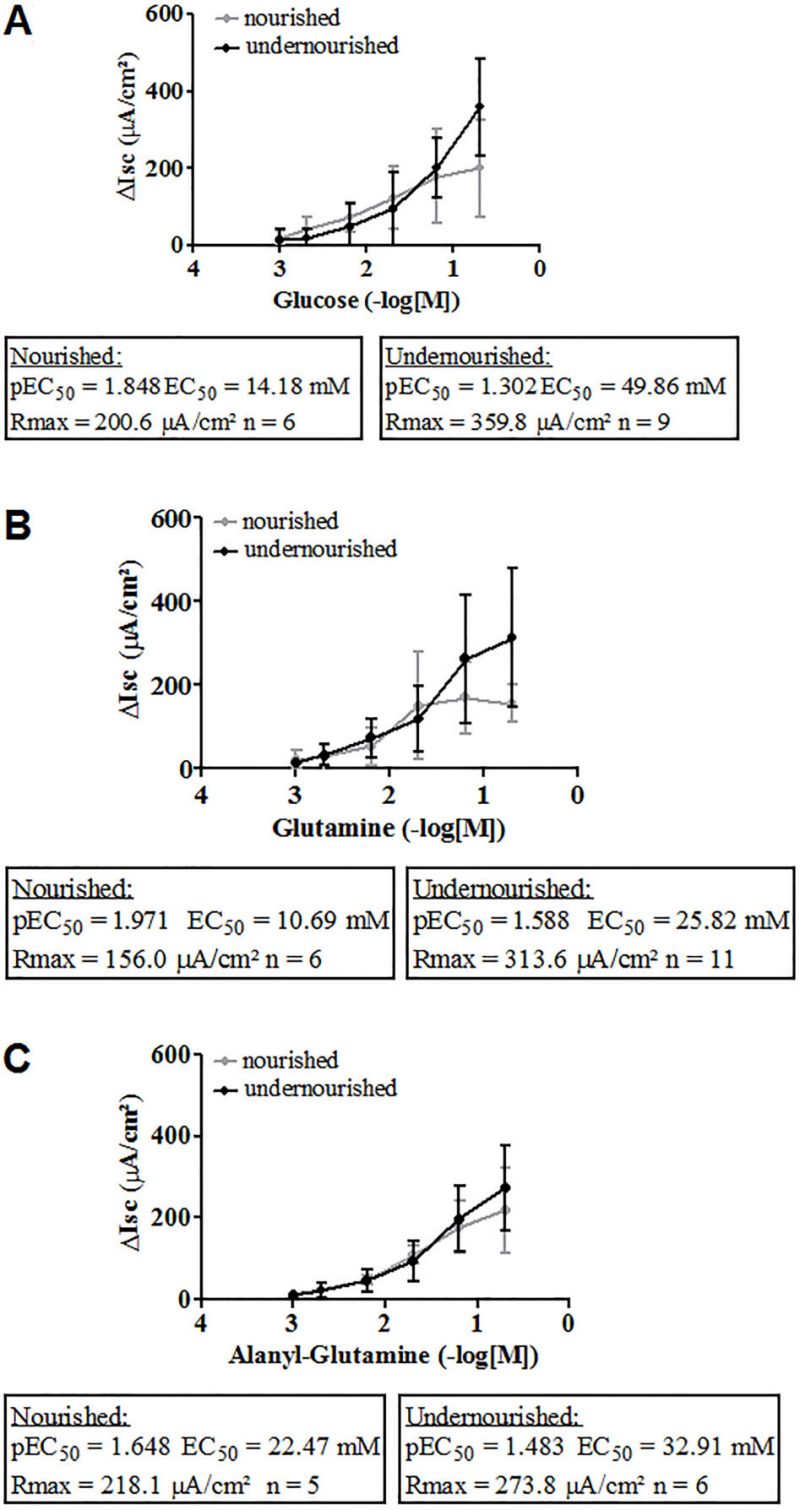
Short-circuit current (I_sc_) measured after cumulative addition of glucose (*A*), glutamine (*B*), and alanyl-glutamine (*C*). Substrates were added at increasing concentrations (1, 2, 7, 20, 70, and 200 mM) at 30 min time intervals with subsequent measurement of I_sc_. Results are reported as means±SE (n = 5-11 Ussing chambers from four mice). P<0.05, maximum responses were significantly different between groups only for the maximum glucose concentration (two-way ANOVA followed by the Bonferroni *post hoc* test).

We plotted the results in a concentration-effect curve analysis for glutamine, alanyl-glutamine and glucose substrates. The correspondent pEC_50_ values for each substrate did not differ between nourished and undernourished groups. Maximum responses were not statistically different between groups, except for the maximum glucose concentration (P<0.05; Figure 5).

### Effect of acute RDB on intestinal transporters and tight junctions transcription, and protein expression

**Figure 6:**
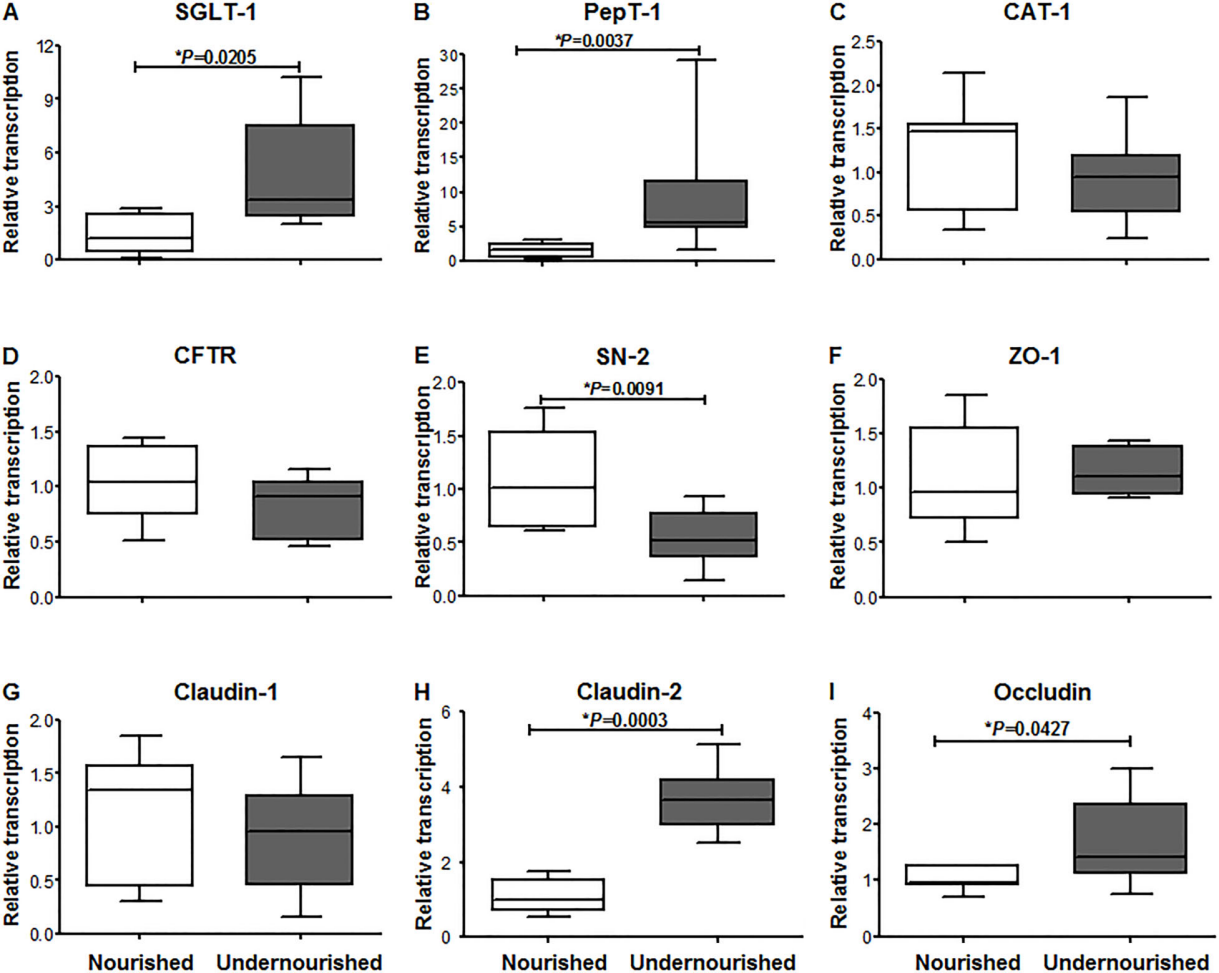
Effects of the regional basic diet on relative expression of *A*, SGLT-1; *B*, PepT-1; *C*, CAT-1; *D*, CFTR; *E*, SN-2; *F*, ZO-1; *G*, claudin-1; *H*, claudin-2; *I*, occludin. *P<0.05, Mann-Whitney test (n=7).

**Figure 7:**
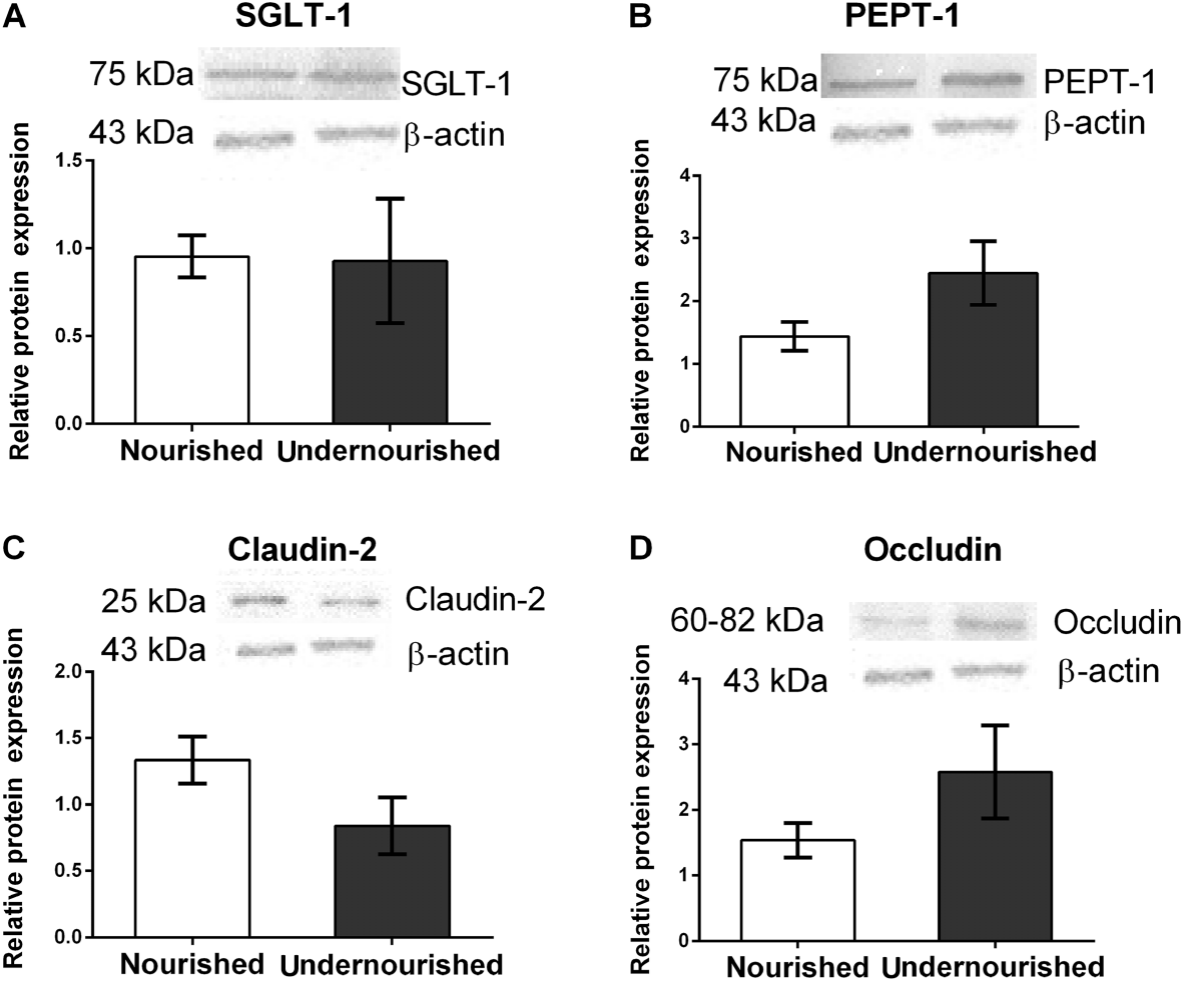
Effect of regional basic diet on protein expression of intestinal transporters and tight junctions. *A*, SGLT-1; *B*, PepT-1; *C*, claudin-2; *D*, occludin. Data are reported as means±SE (n=4). P>0.05, Mann-Whitney test.

The data showed different profiles of transcription of intestinal transports. mRNA transcriptions of SGLT-1 and PEPT-1 were significantly higher in the undernourished group compared to the nourished group (P=0.0205 and P=0.0037, respectively). Conversely, SN-2 transcription was significantly decreased in the animals who received RBD diet (P=0.0091). No changes were found in relative transcriptions of CAT-1 and CFTR. Regarding the effect of RBD diet on mRNA concentration of tight junctions, claudin-2 and occludin transcription, were significantly increased in the undernourished group (P=0.0003 and P=0.0427, respectively; Figure 6). According to the mRNA results, we chose to evaluate the expression of proteins SGLT-1, PEPT, claudin-2 and occludin by western blotting. Unlike differences seen by mRNA analyses, no statistical difference was observed between undernourished and nourished groups for these proteins (Figure 7).

## Discussion

In the present study, we demonstrated in a mouse model that the RBD (regional basic diet, from Northeastern Brazil) promotes undernutrition during a short period of consumption (7 consecutive days), with the objective to understand the early intestinal pathophysiology of undernourished children in developing countries (24,25). In this model, moderate acute undernutrition is characterized by impaired growth, decreased crypt depth with no alterations in villus area, reduced basal I_sc_ and disturbance of gene transcription in intestinal components of the nutrient transport systems in the ileum, with no accompanying changes in protein expression.

Previously, RBD-induced undernutrition has been evaluated only under conditions of chronic exposure, (11,12,26) with incomplete results about the short-term effects of RBD (i.e., no data about the electrophysiological parameters of absorption and their associated mechanisms). In addition, these studies did not evaluate the ileum portion of the small intestine.

RBD-induced undernutrition is characterized mainly by retardation of body weight gain (11,27). Our results demonstrated this decline within the early days of consumption, which continued throughout daily monitoring. This effect might be attributed to the low nutritional value of this diet (protein-, fat-, and mineral-deficient) and hypophagia during the first 2 days of administration, with subsequent growth catch-up. Another study of RBD diet-induced undernutrition observed similar body weight reduction and food ingestion results (12). A multideficient diet with 7.44% protein *vs* a control diet with 20% protein caused malnutrition, reflected by poor growth and development as seen in other mouse models (10,28).

Some studies have shown significant changes in the morphological architecture of microvilli and in the area of intestinal absorption during moderate to long periods of RBD feeding (11,12). Our model of moderate acute undernutrition did not reveal significant differences in the area of the villi, but there was a decrease in the crypt depth. Another study indicated that RBD-induced malnutrition was characterized by decreased crypt depth (12). A recent study of female adult mice fed with a diet similar to RBD also showed no alterations in villus height (13). Cell proliferation and apoptosis are indicated as potential underlying mechanisms for alteration of intestinal morphology by RBD exposure (12). Morphological and functional changes may reflect either beneficial or detrimental effects on nutrient and ion transports as an adaptive mechanism to poor nutrition (29). In a piglet model of short-term protein-energy malnutrition, body weight decreased with no effects on villus height, intestinal permeability or occludin tight junction protein expression (30). Corroborating those findings, these data indicated that short-term undernourished states might lead to slightly altered cell proliferation and apoptosis, without drastic consequences on intestinal morphology or transport.

The small intestine maintains a complex system of transporters with electrophysiological properties necessary to maintain homeostasis associated with paracellular and transcellular fluxes via intestinal epithelial cells (19,31,32). In this study, moderate acute undernutrition was characterized by a reduction in I_sc_ at baseline. Previous studies have shown that the RBD and protein-restricted diets can increase I_sc_ in the jejunum of mice (12,29). Confirming unaltered transporter expression, challenges with glucose, glutamine and alanyl-glutamine substrates showed similar pEC_50_ values between groups. Although the maximum response induced by glucose was different between undernourished and nourished groups, the pEC_50_ is the best measure of receptor/agonist affinity and its potency (33,34). These data suggest a complete responsiveness of the intestinal absorption ability of the undernourished ileum. Furthermore, transepithelial resistance was not altered, which corroborates the unimpaired paracellular transport (29).

In order to maintain membrane stability between epithelial cells in the small intestine, claudins play an important role in the complex intestinal transporters system, contributing for the proper functioning of SGLT-1 and PepT-1 carriers plus several transporters for amino acids (19,35,36). In our study, undernutrition caused diverse regulation on mRNA levels of intestinal transporters and tight junctions: claudin-2, occludin, SGLT-1 and PepT-1 mRNAs were increased while SN-2 mRNA was decreased. Despite the variation on mRNA levels, the protein expression of SGLT-1, PetT-1, claudin-2 and occludin did not vary. The lack of correlation between mRNA and protein levels of intestinal transporters and tight junctions has also been reported in other studies that addressed dietary restrictions in animals (37,14). Furthermore, this study evaluated these parameters in parallel, and it is well known that mRNA and protein production rates of a gene may occur at different time-points (38,39). Moreover, post-transcriptional and translational mechanisms of regulation might help to explain these findings (40). Furthermore, the protein expression results are in agreement with the results of I_sc_-evoked substrates variation, measured in the concentration-effect approach.

It was shown previously that dietary protein restriction in pregnant rats promoted increased transcription and protein expression of intestinal transporter genes (SGLT-1 and PepT-1) in the duodenum of their offspring, but not in the jejunum or ileum (14). Interestingly, removal of luminal nutrition in rats, by applying total parenteral nutrition for induction of intestinal damage, also increased mRNA of some transporter genes, as PEPT-1, although it did not modify CAT1 and SN-2 (15). In addition, a study of maternal undernutrition showed that newborn and weaning piglets did not have altered expression of mRNA for SGLT1 and PEPT-1 (16). Another study reported that in malnourished conditions the localization of SGLT1 does not change (19). In our RBD mouse model, transcriptional levels of SGLT-1 and PEPT-1 transporters were increased in the ileum, with no effects on protein expression. These data highlight the complex regulation of genetic expression of these transporters system (29).

This study has some limitations and perspectives. Biochemical tests, such as oral glucose tolerance test and lipid profile, could have been performed for evaluation of intestinal absorption to reinforce the non-alterations found. Moreover, measuring other physical parameters and behavioral characteristics could provide a more detailed evaluation of animal development. It is also important to point out that, although we were interested in early effects of RBD on intestinal transport, a gap of potential persistent effects of this diet in our model is still evident. Characterizing how the transition from moderate acute undernutrition to severe undernutrition occurs is a question for future studies.

In conclusion, these results demonstrate the early effects of RBD on mice, indicating a state characterized by acute reductions in body weight and ileal crypt depth, without dramatic alterations to villus morphometry, intestinal nutrient transport, transepithelial resistance, and tight junction protein expression. By elucidating the early effects of undernutrition on physiology and in the gut, this study adds important value to the subject by indicating complex adaptive mechanisms of intestinal nutrient transporters and tight junction protein expression along the continuum from acute to chronic states of undernutrition.
